# Impact of surgery and complications on GI recovery after SBO: Insights from the SnapSBO cohort

**DOI:** 10.1111/codi.70411

**Published:** 2026-03-02

**Authors:** Matthew J. Lee, Lewis J. Kaplan, Shahin Mohseni, Matteo Cimino, Hayato Kurihara, Isidro Martinez‐Casas, Gary A. Bass, SnapSBO Collaborators, Gary Alan Bass, Isidro Martínez Casas, Lewis J. Kaplan, Hayato Kurihara, Matthew J. Lee, Shahin Mohseni, Matteo Cimino, Pavel Karasek, Agron Dogjani, Kastriot Subashi, Klevis Doci, Joana Spaho, Ali Abdulla, Sara Ahmed, Yusuf AlAnsari, Mariam AlKooheji, Alaa Marzooq, Khaled Nazzal, Emir Ahmetašević, Zlatan Mehmedović, Maja Kovačević, Jasminka Mujkanović, Marie Peter Svenningsen, Marie Peter Møller, Gitte Emilje Olsen, Abeer Aboalazayem, Muhammad Ashrad Awad, Mahmoud M. A. Elfiky, Moemen Farouk, Mostafa Gad, Basma Magdy, Peep Talving, Edgar Lipping, Edgar Lipping, Sten Saar, Artjom Bahhir, Maarja Talviste, Vincent Dubuisson, Luca Cigagna, Luigi Cayre, Spyridon Christodoulou, Panagiotis Kokoropoulos, Ioannis Margaris, Maria Papadoliopoulou, Theodoros A. Sidiropoulos, Panteleimon Vassiliu, Evangelos Barkolias, Pavlos Georgalis, Theodosios Kantas, Vasiliki Nikolaou, Aristeidis Papadopoulos, Katerina Tata, Stergios Arapoglou, Ioannis Gkoutziotis, Aikaterini Mpratko, Elissavet Symeonidou, Stylianos Kykalos, Nikolaos Machairas, Adam Mylonakis, Panagiotis Sakarellos, Dimitrios Schizas, Michail Vailas, Iraklis Anastasiadis, Parmenion Patias, Koumarelas Konstantinos, Mourtarakos Saradis, Charles Lee, Chloe Spillane, Dylan Viani Walsh, Nadia Walsh, Thomas Noel Walsh, Gabriel Orsi, Andrew Keane, David Kearney, Emma de Sousa, Michael Sugrue, Anne Marie Doyle, Robert Fitzsimmons, Angus J. Lloyd, Mohammad Saad Qasim, Mashood Ahmed, Taylor Jacoby, Michael E. Kelly, Shafagh Khodadi, Paul McCormick, Éanna J. Ryan, Mahmoud M. Salama, Helen Heneghan, Cian Davis, Odhran K. Ryan, Sean T. Martin, Miklosh Bala, Michele Altomare, Stefano P. B. Cioffi, Andrea Spota, Giada Panagini, Laura Benuzzi, Stefania Cimbanassi, Noemi DiFuccia, Stefano Manfroni, Alan Biloslavo, Paola Germani, Nicolo de Manzini, Manuela Mastronardi, Anna Modica, Serena Scomersi, Gabriele Bellio, Luigi Cayre, Gaia Altieri, Pietro Fransvea, Gabriele Sganga, Silvia Tedesco, Francesca Bunino, Sabrina Caspani, Daniele DelFabbro, Simone Giudici, Giulia Mauri, Paolo Meneghesso, Enrico Ortolano, Antonella D’addiego, Francesca Di Vittorio, Gabriele Bormolini, Michele Carlucci, Giovanni Pesenti, Claudia Tintori, Mauro Zago, Martina Zambon, Simona Meneghini, Andrea Mingoli, Giulia Duranti, Gioia Brachini, Mehdi Hanafi, Clara Valdez Cruz, Andrea Alfredo Huerta de León, Jose García Regalado, Pasquale de Jesús Cristiano Nakhal, Diego Enrique Rodríguez González, Jose Ruiz, Salvador Lozada Jimenez, Oscar Carlos Delgado, Monserrat Reyes Zamorano, Anyely Fuertes Muñoz, Ademola Adetoyese Adeyeye, Ehis Afeikhena, Akinola Akinmade, Babatunde Mustapha, Jaroslav Presl, Patrick Rebnegger, Bjoern Rudisch, Gruenfelder Johanna, Rokitte Karin, Filipa M. CorteReal, Jorge A. Pereira, Joao L. Pinheiro, Daniela M. Pinto, Andreia J. Santos, Andreia M. Silva, Susana Henriques, Joao Melo, António Miguel Pereira, Antonio Miguel Pereira, Ana Margarida Cabral, Bruno Dias Couto, Barbara Nunes Gama, Catarina Santos Rodrigues, Mara Nunes, Bruno Ribeiro Silva, Daniela Tavares, Daniela Tavares, Toma Mihai, Oprea C. Valentin, Srdjan S. Putnik, Petar Andkic, Marija Djujic, Rastislav Filko, Vanja Kunkin, Andjela Milak, Aleksandar Ognjenovic, Nebojsa Mitrovic, Goran Aleksandric, Mihailo Bezmarević, Sasa Dragović, Milan Jovanović, Bosko Milev, Miroslav Mitrović, Srdjan Petković, Valentina Isakovic, Nikola Zoran Nikolic, Predrag Radic, Dragan Luka Vasic, Zlatibor M. Loncar, Dusan D. Micic, Vladimir R. Resanovic, Pavle D. Vladimir, Krstina S. Doklestic Vasiljev, Ljiljana Velibor Milic, Vladica Velibor Cuk, Jovan Todor Juloski, Radisav Slavoljub Radulovic, Dragana Dragan Arbutina, Jacobo Trebol, Manuel Torres‐Jurado, Andres J. Valera‐Montiel, Francisco E. Blanco‐Antona, Beatriz de Andrés‐Asenjo, Maria Ruiz‐Soriano, Tania Gómez‐Sanz, Andrea Vázquez‐Fernández, Juan Beltran de Heredia, Cristina Rey‐Valcárcel, Monica Ballón‐Bordon, Maria Pérez‐Díaz, Maria Dolores Sanchez‐Rodriguez, Jose David Gonzalez‐Esteban, Celia Alegre Nevado, Ricardo Montenegro Romero, Andrea Campos‐Serra, Raquel Gracia‐Roman, Heura Llaquet‐Bayo, Anna Muñoz‐Campaña, Giulia Vitiello, Lorena Apodaca Murguiondo, Inigo Augusto Ponce, Amaia Garcia Dominguez, Aintzane Lizarazu Perez, Elena Sagarra Cebolla, Mónica García Aparicio, Paloma Garaulet González, Benito Miguel Josa Martínez, Miriam Fraile Vasallo, M. Morales Meseguer, Mónica MengualBallester, Isabel Andrés Lucas Zamorano, Jose Martinez Moreno, Manuel Luis Buitrago Ruiz, Clara Piñera Morcillo, Alberto Díaz García, Hanna Hernández Oaknin, Maria Pellicer Barreda, Jennifer Amparo García Niebla, Antonio Pérez Álvarez, Diego Cordova, Laura Jiménez, Fernando Mendoza, Cristina Vera, Alberto Vilar Tabanera, María de los Ángeles Gil‐Olarte Márquez, José Antonio López‐Ruiz, Mª. Estela Romero‐Vargas, Julio Reguera‐Rosal, Alberto García‐García, Beatriz Marenco de la Cuadra, Eduardo Perea del Pozo, Virginia Duran Muñoz, Felipe Pareja Ciuró, Ainoa Benavides dos Santos, Ernest Bombuy, Anna G‐Monferrer, Sandra López Gordo, José Guerra, Vanessa Sojo, Begona De Soto, Aaron Roman, Ana María González‐Castillo, Elena Manzo, Estela Membrilla‐Fernandez, Amalia Pelegrina‐Manzano, Simone Cremona, Alexander Forero‐Torres, Santiago Valderrabano, Francisco Reinoso Olmedo, Fuad Lopez Fernandez, Aitor Landaluce‐Olavarria, Jon Barrutia‐ Leonardo, Alba Garcia‐Trancho, Melania Gonzalez‐De Miguel, Izaskun Markinez‐Gordobil, Maryam Makki, Dana Altamimi, Sadhika Vinod, Olga Rutka, John V. Taylor, M. Denton, S. Gourgiotis, R. Ravi, A. J. Ribbits, Jared Wohlgemut, Shehryar Rangana Khan, Christopher Leiberman, Sabreen P. Elbakri, Charlie A. Edgar, Conor Magee, Oluwaseun Oyekan, Mehwish Ansar, Jeremy Wilson, Rahel Rashid, Deborah Atwell, Joshua Cassedy, Brianna Gabriel, William Hoff, Shyam Murali, Anna E. Garcia Whitlock, Carolyn Susman, Sarah Barnett, Emily Ertmann, Camden DeSanctis, Pavel Karasek, Nathan Klingensmith, Dale F. Butler, Brandon Bruns, Ankeeta Mehta, Vanessa Nomellini, Keyus Patel, Anthony Tannous

**Affiliations:** ^1^ Department for Applied Health Research University of Birmingham Birmingham UK; ^2^ Center for Emergency Surgery Outcomes Research University of Pennsylvania Philadelphia Pennsylvania USA; ^3^ Section of Surgical Critical Care, Surgical Services Corporal Michael J. Crescenz Veterans' Affairs Medical Center Philadelphia Pennsylvania USA; ^4^ Division of Traumatology, Surgical Critical Care, and Emergency Surgery Perelman School of Medicine of the University of Pennsylvania Philadelphia Pennsylvania USA; ^5^ Department of Surgery Orebro University Hospital Orebro Sweden; ^6^ Department of Emergency Surgery Fondazione IRCCS Ca′ Granda Ospedale Maggiore Policlinico Milan Italy; ^7^ Unidad de Cirugía de Urgencias y Trauma del Hospital Universitario Virgen del Rocio Sevilla Spain

**Keywords:** electronic health records/integration, gastrointestinal function/recovery, patient‐reported outcome measures/standards, quality of life/surgery, small bowel obstruction/diagnosis

## Abstract

**Background:**

Small bowel obstruction (SBO) is a common surgical emergency associated with impaired gastrointestinal (GI) function and prolonged recovery. The PRO‐diGI patient‐reported outcome measure (PROM) assesses patients' reports on key domains of appetite, nausea, bowel function, well‐being and overall GI function. This study evaluated the influence of demographic and treatment factors on GI recovery following SBO and examined whether these associations persisted after balancing for baseline differences using propensity score matching (PSM).

**Methods:**

An international prospective multicentre cohort study enrolled adult patients undergoing treatment for SBO of any aetiology. GI recovery was assessed using the PRO‐diGI tool. Multivariable regression models were used to identify associations between clinical factors and PROM scores. Regression coefficients (*β*) with 95% confidence intervals were calculated. PSM was performed within the adhesional SBO subgroup to minimize confounding from differences in follow‐up time and baseline characteristics.

**Results:**

Of 1734 participants, 644 completed all PROM domains. Among patients contributing PROM data, surgical intervention was associated with improved nausea (*β* 5.9, 95% confidence interval 1.1–11.0) and overall GI function (*β* 6.8, 95% confidence interval 0.54–13.0) scores. Complications were linked to worse nausea (*β* −9.3, 95% confidence interval −17.0 to −1.7), well‐being (*β* −17.0, 95% confidence interval −29.0 to −4.3), and overall function (*β* −12.0, 95% confidence interval −22.0 to −1.4). Previous nonoperative SBO episodes were associated with reduced appetite scores (*β* −7.3, 95% confidence interval −13.0 to −1.7). In the adhesion PSM cohort, overall GI function remained higher after surgery, and laparoscopic adhesiolysis was associated with superior appetite and overall function scores.

**Discussion:**

Surgical treatment without complications was associated with improved patient‐reported GI recovery after SBO. Persistence of these associations following matching indicates that patient‐reported GI recovery differs across treatment pathways in selected patients, supporting the feasibility and discriminatory value of PRO‐diGI as a patient‐centred outcome measure.

**What does this paper add to the literature?**

This study demonstrates the association between treatment strategies for small bowel obstruction and gastrointestinal PROM outcomes. Models suggest that surgical treatment is associated with improved gastrointestinal recovery, and laparoscopic surgery is associated with improved PROMS vs. open surgery.

## INTRODUCTION

Small bowel obstruction (SBO), which is frequently caused by adhesions, hernias, or cancers, accounts for almost 250,000 deaths and more than 7 million disability adjusted life years globally in 2019 [1, 2]. Typical manifestations, including abdominal pain, vomiting and obstipation, reflect transient intestinal failure (Type I) [3]. Postsurgical outcomes demonstrate substantial morbidity following SBO management [4]. A recently developed SBO core outcome set highlighted the need for patient‐reported outcome measures (PROMs) to assess how different approaches influence patient‐centred care [5]. This core outcome set was developed to standardize the variable metrics that characterize outcome reporting [4].

Efforts to standardize outcome reporting in SBO highlight the persistent absence of patient‐centred endpoints. Patient‐reported outcomes (PROs), directly reported by patients, capture aspects of recovery not apparent to clinicians and which are essential to evaluating treatment effectiveness from the patient perspective. A PROM is the tool or instrument used to report PROs [6]. These may be generic or disease or treatment specific. The PRO‐diGI PROM is a condition‐specific tool developed to assess recovery of gastrointestinal (GI) function following postoperative ileus or intestinal obstruction management. Developed through qualitative inquiry and psychometric validation, it quantifies four domains: appetite, nausea, bowel function and well‐being, and also provides an overall global rating of GI function. Its development addressed priorities identified in international consensus processes on postoperative ileus and SBO. The tool demonstrates strong face validity, test–retest reliability, and responsiveness [7].

To date, PROM data following SBO treatment are scarce, and few studies explore how patient or treatment factors influence subjective GI recovery. The present study aimed to examine associations between demographic and treatment characteristics, including operative approach and complications, and patient‐reported GI recovery after SBO, and to determine whether these associations persist after adjustment for baseline differences. Beyond examining associations between patient and treatment characteristics and GI recovery, this study aimed to evaluate whether a condition‐specific PROM could capture dimensions of recovery not reflected by traditional clinical outcomes following SBO.

## METHODS

### Study design

This multi‐centred, nonrandomized, prospective snapshot audit cohort study, SnapSBO, an official project of the European Society for Trauma and Emergency Surgery (ESTES), examined epidemiological and management factors in SBO across varied clinical settings [8]. Data were collected over 6 months (1 November 2023 to 31 May 2024) from consecutively admitted patients with SBO.

### Setting

Hospitals providing emergency surgical care, regardless of geographic location, were eligible to participate. Sites had to register with the study team and secure patient protection approval prior to participation.

### Patient eligibility

Adult patients (≥16 years) with radiologically confirmed mechanical SBO, defined as an obstructive process impeding luminal flow, were included. Functional disorders, such as paralytic ileus without mechanical obstruction, were excluded. Patients were monitored from hospital admission through a 60‐day postdischarge period.

### Approvals

The study protocol was registered with ClinicalTrials.gov (registration ID: NCT05843097). Due to the international nature of the study, sites were required to secure local IRB or governance approvals prior to participation in the study. Each centre complied with local institutional review board approvals, and data governance complied with the European Union General Data Protection Regulation (GDPR; (EU) 2016/679) and Health Insurance Portability and Accountability Act (HIPAA; 1996) data‐protection legislation.

### Outcomes

The primary outcome of interest was PROM values at follow‐up posttreatment, measured using the PRO‐diGI tool. Additional clinical data of interest include treatment strategies (operation/conservative management) and key treatment outcomes, including surgical intervention and approach, and surgical complications.

### Data collection and management

Data were gathered through daily reviews of health records, paper‐based logs and records from emergency departments, admission lists and operating rooms. Local staff entered data into REDCap (version 14.8.3 ‐ © 2024 Vanderbilt University) with mandatory fields to minimize missing data. As this was an unfunded, international snapshot, data collection relied on local investigator engagement and routine clinical workflows, introducing potential for missing or inaccurate data. PROM completion depended on successful outpatient follow‐up and patient engagement.

Where participants were followed up in an outpatient clinic following completion of their hospital stay, PRO‐diGI responses were captured. PRO‐diGI contains 15 items across four domains: Appetite, Nausea, Bowel function and Well‐being, as well as a global anchor question about self‐reported scalar rating of GI function (0–100; higher scores are better). Each domain is scored by patients using a previously defined process, and domain scores are calculated as a percentage of the total possible score [7]. The best score for a domain is 100, and the worst score is 0. PROMS were completed at follow‐up by the patient, but may have also included support from the clinical team. The instructions of the tool ask participants to recall the prior 7 days.

Descriptive statistics were collated for the entire population, and characteristics of those with and without any PROM data were compared. Those with a complete PROM dataset (complete responses for all domains and overall rating of bowel function) were selected for further analysis. Multivariable models were generated for each domain and overall bowel function. This included demographic factors that might be associated with decisions to manage operatively, factors identified as relevant in previous studies [1, 9], and those related to operative intervention.

A subgroup analysis was undertaken for the adhesion subgroup, as this was anticipated to be the largest group that is also accompanied by unique management features. This was used to explore the association of different treatment strategies with PROM‐based outcomes.

To explore the robustness of primary findings to baseline imbalance and variation in follow‐up timing, a prespecified propensity score‐matched (PSM) sensitivity analysis was undertaken within the adhesional SBO subgroup. Propensity scores were estimated using logistic regression, with surgical intervention (operation versus no operation) or operative approach (laparoscopic versus open) as the treatment variable. Covariates included age, sex, the presence of any medical comorbidities (yes/no), and follow‐up duration, the latter incorporated to minimize bias arising from later outpatient review after surgery. Nearest‐neighbour matching without replacement and a 1:1 ratio was used with a calliper of 0.2 standard deviations of the logit of the propensity score. Balance was assessed using standardized mean differences, with values <0.1 considered acceptable. Matched cohorts were then compared using adjusted linear models for each PRO‐diGI domain and the overall GI function score, with follow‐up time retained as a covariate in all comparisons to ensure further adjustment for residual time‐related effects. Regression models were applied within matched cohorts to adjust for residual differences in follow‐up duration and to improve the precision of estimated associations, consistent with recommended analytical practice in matched observational studies.

All analyses were conducted in R (version 4.1.2), with significance set at *p* < 0.05. Models are reported as *β* coefficients with 95% confidence intervals.

## RESULTS

**FIGURE 1:**
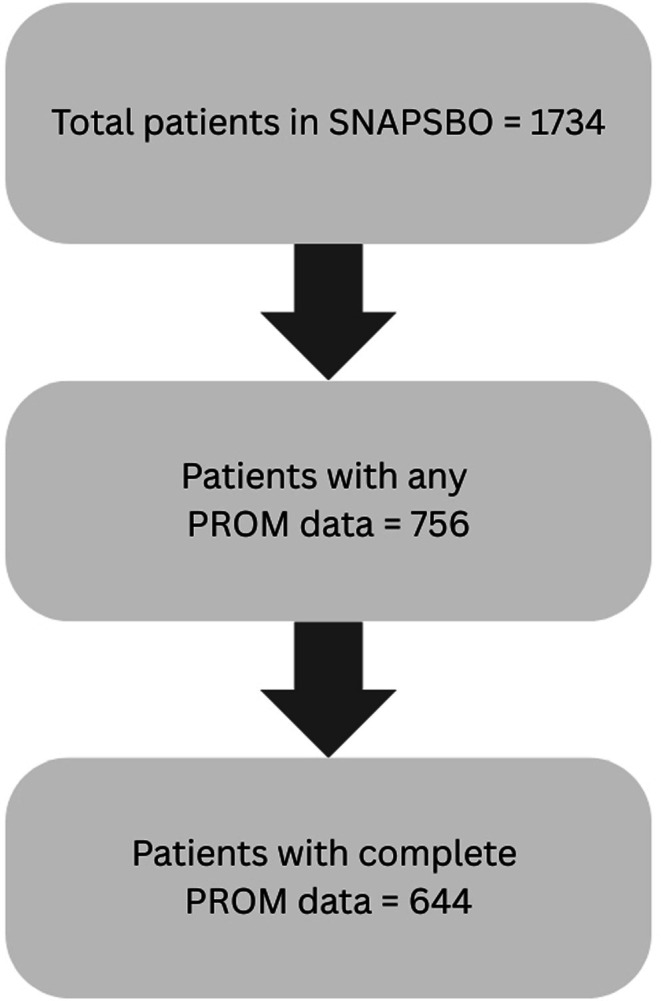
STROBE flowchart. PROM, patient‐reported outcome measure.

Of the 1734 patient episodes recorded, 756 (44%) had any PROM data available, and 644 (37%) provided complete responses across all PRO‐diGI domains. PROM completion was higher among patients who underwent surgery (62% vs. 50%, *p* < 0.001), reflecting differences in outpatient follow‐up pathways. Characteristics of patients with and without complete PROM data are shown in Tables 1 and S1. A STROBE flowchart is presented in Figure 1. PROM data capture was higher among patients who underwent surgery (62% vs. 50%, *p* < 0.001).

**TABLE 1:** Participants with complete PROM data.

Characteristic	Overall *N* = 1737^1^	Incomplete *N* = 1093^1^	Complete *N* = 644^1^	*p*‐value
Age (years)	69.0 (54.0, 80.0)	70.0 (53.0, 80.0)	68.0 (56.0, 79.0)	0.93
Male sex	834 (48%)	525 (48%)	309 (48%)	0.44
Adhesions	982 (57%)	622 (57%)	360 (56%)	0.68
Surgical intervention for SBO	887 (56%)	479 (50%)	408 (64%)	<0.001
Bowel Ischaemia	233 (29%)	126 (29%)	107 (28%)	0.78
Prior nonoperative management	369 (66%)	244 (67%)	125 (63%)	0.35

^1^ Median (Q1, Q3); *n* (%).

Among the 644 with complete PROM data, the median age was 68 years (i.q.r. 56–79), and 48.0% were male. Adhesions accounted for 56.0%, malignancy for 17.0% and hernia‐related obstruction for 9.0%. Surgery was performed in 63.4% of patients; among these, 28.0% experienced postoperative complications and 12.0% underwent laparoscopy.

### Aetiology

**FIGURE 2:**
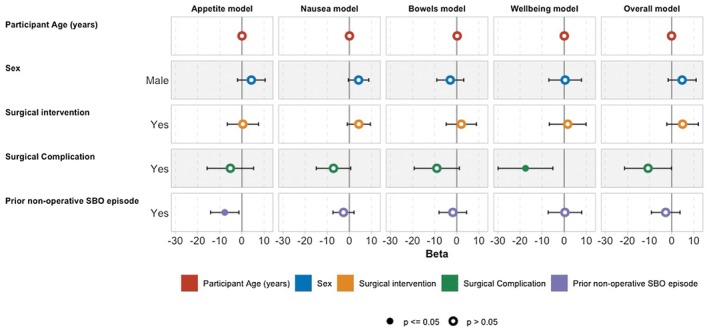
Adjusted associations between patient demographics, treatment characteristics, and PRO‐diGI domain scores. Demographic variables (comorbidity, sex, aetiology) demonstrated limited independent association with patient‐reported outcomes compared with treatment‐related factors. SBO, small bowel obstruction.

In adjusted multivariable analyses, demographic characteristics—including age, sex and SBO aetiology—were not independently associated with differences in PRO‐diGI domain scores or overall GI function (Figure 2; Table S2). In contrast, treatment‐related factors, including operative intervention, complications and operative approach, demonstrated consistent associations with patient‐reported recovery outcomes.

In these models, surgical intervention was associated with higher nausea (*β* 5.9, 95% confidence interval 1.1–11.0, *p* = 0.047) and overall GI function (*β* 6.8, 95% confidence interval 0.5–13.0, *p* = 0.018) scores. Postsurgical complications were independently associated with worse nausea (*β* −9.3, 95% confidence interval −17.0 to −1.7, *p* = 0.010), well‐being (*β* −17.0, 95% confidence interval −29.0 to −4.3, *p* = 0.003) and overall function scores (*β* −12.0, 95% confidence interval −22.0 to −1.4, *p* = 0.012). A history of prior nonoperative SBO episodes was associated with lower appetite (*β* −7.3, 95% confidence interval −13.0 to −1.7, *p* = 0.047). No other demographic or aetiological variables were associated with PROM differences (Figure 2, Table S2).

### Subgroup exploration

Adhesional SBO represented the largest subgroup (982 of 1734, 57%). In this cohort, patterns of PROMs were similar to the overall population, with higher domain scores in surgically managed patients without complications. Use of water‐soluble contrast was not associated with differences in any PRO‐diGI domains or overall GI function in the adhesional SBO subgroup (Table S3). Domain scores for appetite, nausea, bowel function and well‐being were comparable between patients who did and did not receive contrast. Laparoscopy was undertaken in a subset of patients and was associated with higher appetite scores compared with open surgery (median 90 versus 85, *p* = 0.026).

### Sensitivity analysis using propensity score matching in adhesive SBO

To account for potential confounding by follow‐up timing and baseline characteristics, a propensity score‐matched analysis was performed within the adhesional SBO subgroup.

### Nonoperative versus operative management

Matching improved balance across groups, but all showed a standardized mean difference >0.1. After matching (*n* = 286 pairs, Table S4, Figure S1). There were no differences in appetite, nausea, bowel or well‐being between nonoperative and operative groups (Table 2).

**TABLE 2:** Effect on PROM outcomes for operative vs. nonoperative management.

Outcome	Adjusted difference (95% CI)	*p*‐value
Appetite	−0.44 (−5.34, 4.46)	0.860
Nausea	0.61 (−3.33, 4.55)	0.761
Bowels	−0.31 (−4.98, 4.36)	0.897
Well‐being	−1.51 (−7.45, 4.44)	0.618
Overall bowel function	4.25 (−0.26, 8.75)	0.065

### Laparoscopic versus open surgery

Matching was achieved in groups comparing surgery, where standardized mean difference was <0.1 across all variables (*n* = 94 pairs, Table S5, Figure S2), laparoscopy was associated with higher appetite (mean difference 11.3, 95% confidence interval 3.1–19.5, *p* = 0.007) and overall GI function (mean difference 9.58, 95% confidence interval 3.6–15.6, *p* = 0.002) scores (Table 3).

**TABLE 3:** Effect on PROM outcomes for laparoscopic vs. open surgical treatment.

Outcome	Adjusted difference (95% CI)	*p*‐value
Appetite	11.28 (3.09, 19.47)	0.007
Nausea	3.30 (−3.69, 10.29)	0.350
Bowels	5.47 (−2.56, 13.50)	0.179
Well‐being	9.04 (−1.24, 19.32)	0.084
Overall bowel function	9.58 (3.61, 15.55)	0.002

## DISCUSSION

This international cohort provides an empirically grounded account of patient‐reported GI recovery following SBO, measured using a condition‐specific and validated tool [10]. By applying PRO‐diGI at scale across an international cohort, the study demonstrates that uncomplicated surgical treatment, particularly laparoscopic adhesiolysis, is associated with higher patient‐reported recovery scores, whereas postoperative complications and prior nonoperative episodes correspond to worse outcomes. These associations persisted after adjustment for baseline differences, supporting a potential causal link between effective operative resolution and more complete recovery of GI function.

Although water‐soluble contrast is widely used to aid diagnosis and predict short‐term resolution of adhesive SBO, we did not observe an association with patient‐reported GI recovery at follow‐up. This suggests that while contrast may influence early management decisions and length of stay, it may not translate into differences in longer‐term patient‐perceived functional recovery. This distinction highlights the complementary roles of traditional clinical endpoints and PROM‐based outcomes.

Notably, demographic factors showed minimal independent association with patient‐reported recovery, suggesting that PRO‐diGI is more sensitive to treatment‐related experiences than to baseline patient characteristics. The association between uncomplicated surgery and higher patient‐reported recovery scores reflects differences in patient‐perceived GI function within selected treatment pathways, rather than evidence that surgery represents a universally superior or more complete form of recovery. In patients with complex or extensive adhesive disease, nonoperative management may appropriately prioritize avoidance of operative risk, with acceptance of persistent low‐grade symptoms. Interpretation of PROM differences must therefore be situated within the clinical context in which treatment decisions are made.

The observed association between laparoscopic surgery and superior patient‐reported recovery should be interpreted in the context of case selection. Laparoscopy in adhesional SBO is typically reserved for patients with limited adhesive burden and favourable anatomy, whereas dense or complex adhesions often necessitate open surgery. Although propensity score matching improved balance for measured variables, residual confounding related to disease severity and operative complexity is likely and cannot be excluded. It is notable that the association of laparoscopic surgery with better PROMs vs. open surgery reflects the findings of the LASSO trial [10]. However, the deleterious influence of postoperative complications on well‐being and global GI function indicates that adverse events can attenuate these perceived benefits. This is congruent with previous work using the Euro‐Qol 5D‐5L tool, demonstrating that surgical complications are associated with negative quality of life and loss of quality‐adjusted life years [11].

The association between prior nonoperative SBO episodes and poorer appetite scores might reflect underlying disease complexity rather than a causal effect of conservative management. Patients managed nonoperatively may have extensive or recurrent adhesive disease, where surgical intervention is deferred because of high operative risk until symptoms become unavoidable. In this context, persistent low‐gradeGI symptoms may represent an accepted trade‐off rather than treatment failure. This highlights the importance of interpreting PROM differences in SBO within the context of clinical decision‐making and case selection. An alternative theory is that the association with reduced appetite may reflect persistent low‐grade adhesive disease or altered neurohormonal signalling that affects feeding behaviour.

A central finding of this study is that PRO‐diGI captures dimensions of GI symptom burden and recovery trajectory that are not apparent through traditional clinician‐reported outcomes, such as complications, length of stay or radiological resolution. This distinction is particularly relevant in SBO, where clinical success does not necessarily equate to patient‐perceived recovery. By embedding the PRO‐diGI instrument within a large, prospective cohort, this study expands outcome assessment beyond morbidity and mortality to incorporate patient experience. A key methodological refinement of this study was the addition of a propensity score‐matched analysis focused on adhesive small bowel obstruction. The propensity score‐matched analyses were intended to test the robustness of observed associations rather than to provide causal estimates. By addressing baseline imbalance and follow‐up timing, these analyses support, but do not supersede, the primary multivariable findings, and residual confounding related to disease severity and operative complexity remains possible. The persistence of findings after matching suggests that patient‐perceived recovery differs across treatment pathways even after adjustment for measured baseline differences, without implying that uncomplicated surgery is universally achievable or appropriate. Accordingly, these findings should be interpreted as descriptive of patient‐reported recovery within real‐world care pathways rather than as guidance on optimal management.

That a PROM tool was readily utilized across an international cohort during outpatient follow‐up raises the question of feasibility for use in research and quality‐improvement settings. Routine clinical use would require additional validation, including assessment of longitudinal responsiveness, cross‐cultural adaptation and integration into clinical workflows. In the interim, PRO‐diGI may provide a useful outcome measure for comparative effectiveness research, audit and trial design in SBO. Acquiring objective data regarding the patient's perception of their care and its outcomes is invaluable in shaping conversations during the visit and alerting the clinician to perceived inadequacies so that they may be readily addressed. Embedding such tools in the outpatient space would also provide a ready means of tracking patient experiences that is distinct from commonly utilized tools, such as the US Press‐Gainey score, which is condition‐agnostic and is also uninformative during a patient visit, as it is anonymous [12]. While the ProDiGi tool was not altered based on cultural or language differences, such refinements are readily envisionable.

These findings establish the feasibility of integrating PROs into international surgical registries and suggest that PROM data may provide additional resolution for evaluating treatment quality [11]. With further longitudinal validation, PRO‐diGI could inform trial design by defining patient‐centred endpoints and enabling estimation of the minimum clinically important difference (MCID). Such data may, in turn, help clarify whether earlier operative intervention confers not only anatomical resolution but also improved functional recovery. This will also permit calculation of a MCID for the tool in SBO [13]. This may permit a trial design with a PROM as the primary outcome. Longer term follow‐up may also allow assessment of the prognostic validity of the tool, identifying those at risk of early recurrence of obstruction. Work on long‐term appetite and gut hormone signalling in nonoperatively managed SBO might also be considered. Our findings, therefore, provide the conceptual foundation for PROM‐based endpoints in future clinical trials and can inform sample‐size calculations, intervention design and interpretation of recovery trajectories.

Study findings must be placed in the context of the limitations. Only a subset of enrolled patients contributed complete PROM data, introducing potential selection bias. Patients undergoing surgery were more likely to complete follow‐up PROMs, likely reflecting structured postoperative review, whereas conservatively managed patients may have been discharged without routine follow‐up. This means that missing PROM data might be a signal of system‐level follow‐up heterogeneity rather than a data quality failure. As a result, findings should be interpreted as associations among patients engaged in follow‐up rather than as population‐level estimates of recovery. The direction of bias is therefore likely towards patients with more favourable recovery trajectories and greater healthcare engagement. Only follow‐up scores were collected, and not baseline scores, precluding assessing changes in scores from baseline; a difficult task, as baseline score capture requires identifying patients prior to presentation for emergency care. However, the study has analysed scores as groups according to treatment strategy, assessing averaged scores for a single timepoint. As with all large datasets, despite extensive data, clinical inaccuracies remain a potential issue. PRO‐diGI, formulated in the United Kingdom, is currently only available in English, introducing potential language and culture bias. Because not all participating countries utilize English, cross‐cultural validity and interpretation accuracy are reasonable questions to raise [14]. Nonetheless, our approach is pragmatic and highlights the potential need for the tool to be translated into additional languages. Patients treated with surgery account for 2/3 of the study population, suggesting bias in follow‐up favouring those who underwent an operation for SBO. The data is also limited by the snapshot audit approach. There are additional granular management or outcomes aspects that would be valuable, but were not captured for pragmatic reasons. Approaches to follow‐up are not routine, and there may be responder bias as well. Due to the small number of nonadhesion aetiologies followed up, it was not possible to adjust for these in assessment models. However, the internal consistency and clinical plausibility of the findings suggest that our data provide meaningful insights into patient experiences during SBO recovery despite these potential limitations. The incorporation of PSM reduces, but does not eliminate, the possibility of residual confounding, and prospective designs with embedded PROM collection will ultimately be required to define causal relationships.

There are implications for policymakers raised by this study. With further data, surgeons could consider how PROMs should influence guidelines for best practice. Most evidence‐informed expert consensus guidelines, such as the WSES Bologna guidelines for the management of adhesive SBO, predominantly focus on homogenizing clinical rather than patient‐centred outcomes [15]. Incorporation of PROM‐based endpoints could complement these by quantifying the patient's recovery experience and allow tailoring of postdischarge support [16]. Furthermore, they may drive up the standard of care [17] and support benchmarking across institutions [18]. PROM integration within pragmatic registry frameworks, such as the National Emergency Laparotomy Audit [19], therefore represents a practical step towards a more comprehensive understanding of recovery after SBO or other conditions of interest. Our findings, therefore, provide the conceptual foundation for PROM‐based endpoints in future clinical trials and can inform sample‐size calculations, intervention design and interpretation of recovery trajectories.

## CONCLUSIONS

In this international cohort, uncomplicated and particularly laparoscopic surgery for SBO was associated with superior patient‐reported GI recovery. Complications and prior nonoperative episodes were associated with poorer functional outcomes. The persistence of these associations after adjustment suggests that operative management may confer advantages in functional recovery. These data support the continued evaluation of PROMs, such as PRO‐diGI, into research and quality‐improvement initiatives addressing SBO. The persistence of these associations after propensity matching provides evidence that uncomplicated, and particularly laparoscopic, surgery is associated with higher patient‐reported GI recovery in selected patients. These data support the continued incorporation of PROMs, such as PRO‐diGI, into research and quality‐improvement initiatives addressing SBO. Importantly, this study demonstrates the added value of condition‐specific PROMs in revealing aspects of recovery that would otherwise remain invisible in routine clinical datasets.

## AUTHOR CONTRIBUTIONS

GAB, LJK and MJL conceived the idea, drafted the manuscript and participated in its critical review and final approval. IMC, SM, MC and HK participated in its critical review and final approval. Authors are parsed into the following groups at the end of the manuscript, according to the CREDiT taxonomy: Manuscript Writing Group, SNAPSBO Steering Committee and Study Collaborators and their affiliations.

## FUNDING INFORMATION

No financial support or funding was received for the presented work.

## CONFLICT OF INTEREST STATEMENT

As an employee of the Veterans Health Administration, Dr. Kaplan's opinions do not reflect the opinions or policies of the Veterans Health Administration or the United States Federal government. The other authors have no conflicts of interest to disclose.

## ETHICS STATEMENT

Ethical (e.g. IRB/REC) and/or governance approvals were secured by participating sites in line with local guidance for participation in snapshot studies.

## Supporting information


**Table S1.** Comparison of population with any PROMS vs. no PROM data.
**Table S2**. Comparison of population with any PROMS vs. no PROM data.
**Table S3**: Summary of characteristics and scores according to water soluble contrast receipt.
**Table S4**: Characteristics of matched groups comparing operative vs. non‐operative management of adhesional SBO.
**Table S5**: Characteristics of matched groups comparing open vs. laparoscopic management of adhesional SBO.
**Figure S1**: Love plot for operation vs. no operation matching.
**Figure S2**: Love plot for laparoscopic vs. open operation.

## Data Availability

Research data are not shared.
