# Supplementary material for: Impact of surgery and complications on GI recovery after SBO: Insights from the SnapSBO cohort
Source: Colorectal Dis. 2026 Mar 2;28(3):e70411. doi: 10.1111/codi.70411 (PMC12953193; doi:10.1111/codi.70411)

**Impact of Surgery and Complications on GI Recovery After SBO: Insights from the SnapSBO Cohort**

**Matthew J Lee^1,2^, Lewis J Kaplan^2,3,4^, Shahin Mohseni^5^, Matteo Cimino^6^, Hayato Kurihara^6^, Isidro Martinez-Casas^7^, Gary A Bass^2,4^ *for the SnapSBO Collaborators^.***

1. **Department for Applied Health Research, University of Birmingham, Birmingham, United Kingdom.**
2. **Center for Emergency Surgery Outcomes Research, University of Pennsylvania, Philadelphia, PA, USA.**
3. **Corporal Michael J. Crescenz Veterans’ Affairs Medical Center; Surgical Services, Section of Surgical Critical Care; Philadelphia, PA; USA**
4. **Perelman School of Medicine of the University of Pennsylvania; Division of Traumatology, Surgical Critical Care, and Emergency Surgery, Philadelphia, PA, USA.**
5. **Department of Surgery, Orebro University Hospital, Orebro, Sweden.**
6. **Department of Emergency Surgery, Fondazione IRCCS Ca’ Granda Ospedale Maggiore Policlinico, Milan, MI, Italy.**
7. **Unidad de Cirugía de Urgencias y Trauma del Hospital Universitario Virgen del Rocio, Sevilla, Spain.**

**Corresponding Author:**

Matthew J. Lee, MBChB, PhD, FRCS

BHP Clinician Scientist & Consultant Colorectal Surgeon

Department of Applied Health Sciences, University of Birmingham, Birmingham, United Kingdom.

Email: m.j.lee.1@bham.ac.uk

ORCID: 0000-0001-9971-1635

**Supplementary Materials - Index**

| **Supplementary Methods** |  |
| --- | --- |
| Collaborator list | *page 3* |
| **Supplementary Figures and Tables** |  |
| Table S1 Comparison of population with any PROMS vs no PROM data | *page 6* |
| Table S2 Comparison of population with any PROMS vs no PROM data  Table S3: Summary of characteristics and scores according to treatment group  Table S4: Summary of Summary of characteristics and scores according to water soluble contrast receipt. | *page 7*  *page 8*  *page 9* |

**Supplementary Methods**

**Steering Group Members**

**United States of America:** *University of Pennsylvania, in Philadelphia, PA.* Gary Alan Bass.

**Spain:** *Hospital Universitario Virgen del Rocio in Sevilla, Spain.* Isidro Martínez Casas.

**United States of America:** *University of Pennsylvania, in Philadelphia, PA.* Lewis J Kaplan.

**Italy:** *Fondazione IRCCS Ca’ Granda Ospedale Maggiore Policlinico in Milan, Italy.* Hayato Kurihara.

**United Kingdom:** *University of Birmingham.* Matthew J Lee.

**United Arab Emirates:** *Sheikh Shakhbout Medical City in Abu Dhabi.* Shahin Mohseni.

**Italy:** *Fondazione IRCCS Ca’ Granda Ospedale Maggiore Policlinico in Milan.* Matteo Cimino.

**Database and statistical assistance**

**United States of America:** *University of Pennsylvania, in Philadelphia, PA.* Pavel Karasek

**Center Contributors**

**Albania:** *University Hospital of Trauma.* Agron Dogjani, Kastriot Subashi, Klevis Doci, Joana Spaho.

**Bahrain:** *Salmaniya Medical Complex.* Ali Abdulla, Sara Ahmed, Yusuf AlAnsari, Mariam AlKooheji, Alaa Marzooq, Khaled Nazzal.

**Bosnia and Herzegovina:** *University clinical centre.* Emir Ahmetašević, Zlatan Mehmedović, Maja Kovačević, Jasminka Mujkanović.

**Denmark:** *Nordsjællands Hospital - University of Copenhagen.* Peter Svenningsen, Marie Peter Møller, Gitte Emilje Olsen.

**Egypt:** *Cairo University.* Abeer Aboalazayem, Muhammad Ashrad Awad, Mahmoud MA Elfiky, Moemen Farouk, Mostafa Gad, Basma Magdy.

**Estonia:** *North Estonia Medical Centre.* Peep Talving, Edgar Lipping, Edgar Lipping, Sten Saar, Artjom Bahhir, Maarja Talviste.

**France:** *Centre Hospitalier Universitaire de Bordeaux.* Vincent Dubuisson, Luca Cigagna, Luigi Cayre.

**Greece:** *Attikon University Hospital.* Spyridon Christodoulou, Panagiotis Kokoropoulos, Ioannis Margaris, Maria Papadoliopoulou, Theodoros A Sidiropoulos, Panteleimon Vassiliu. *General Hospital of Nikaia.* Evangelos Barkolias, Pavlos Georgalis, Theodosios Kantas, Vasiliki Nikolaou, Aristeidis Papadopoulos, Katerina Tata. *Ippokrateio General Hospital.* Stergios Arapoglou, Ioannis Gkoutziotis, Aikaterini Mpratko, Elissavet Symeonidou. *Laikon General Hospital.* Stylianos Kykalos, Nikolaos Machairas, Adam Mylonakis, Panagiotis Sakarellos, Dimitrios Schizas, Michail Vailas. *Nafplio General Hospital.* Iraklis Anastasiadis, Parmenion Patias, Koumarelas Konstantinos, Mourtarakos Saradis.

**Ireland:** *Beaumont Hospital.* Charles Lee, Chloe Spillane, Dylan Viani Walsh, Nadia Walsh, Thomas Noel Walsh. *Connolly Hospital Blanchardstown.* Gabriel Orsi, Andrew Keane, David Kearney, Emma de Sousa. *Letterkenny University Hospital.* Michael Sugrue, Anne Marie Doyle, Robert Fitzsimmons, Angus J Lloyd, Mohammad Saad Qasim, Mashood Ahmed. *St James Hospital.* Taylor Jacoby, Michael E Kelly, Shafagh Khodadi, Paul McCormick, Éanna J Ryan, Mahmoud M Salama. *St Vincents University Hospital.* Helen Heneghan, Cian Davis, Odhran K Ryan, Sean T Martin.

**Israel:** *Hadassah Medical Center and Faculty of Medicine, Hebrew University of Jerusalem.* Miklosh Bala.

**Italy:** *ASST Grande Ospedale Metropolitano Niguarda.* Michele Altomare, Stefano PB Cioffi, Andrea Spota, Giada Panagini, Laura Benuzzi, Stefania Cimbanassi. *Azienda Ospedaliera San Camillo Forlaninin.* Noemi DiFuccia, Stefano Manfroni. *Cattinara University Hospital.* Alan Biloslavo, Paola Germani, Nicolo de Manzini, Manuela Mastronardi, Anna Modica, Serena Scomersi. *Fondazione IRCCS Ca’ Granda Ospedale Maggiore Policlinico.* Gabriele Bellio, Luigi Cayre. *Fondazione Policlinico Universitario A Gemelli IRCCS.* Gaia Altieri, Pietro Fransvea, Gabriele Sganga, Silvia Tedesco. *IRCCS Humanitas Research Hospital.* Francesca Bunino, Sabrina Caspani, Daniele DelFabbro, Simone Giudici, Giulia Mauri, Paolo Meneghesso. *IRCCS Ospedale San Raffaele.* Enrico Ortolano, Antonella D'addiego, Francesca Di Vittorio, Gabriele Bormolini, Michele Carlucci. *Ospedale Alesandro Manzoni.* Giovanni Pesenti, Claudia Tintori, Mauro Zago. *Policlinico Umberto I.* Martina Zambon, Simona Meneghini, Andrea Mingoli, Giulia Duranti, Gioia Brachini, Pierfrancesco Lapolla. *University Hospital.* Mehdi Hanafi.

**México:** *Nuevo Hospital Civil de Guadalajara "Dr.* Juan I. Menchaca". Clara Valdez Cruz, Andrea Alfredo Huerta de León, Jose García Regalado, Pasquale de Jesús Cristiano Nakhal, Diego Enrique Rodríguez González. *Hospital Regional de Alta Especialidad del Bajío.* Jose Ruiz, Salvador Lozada Jimenez, Oscar Carlos Delgado, Monserrat Reyes Zamorano, Anyely Fuertes Muñoz.

**Nigeria:** *Afe Babalola University Multisystem Hospital.* Ademola Adetoyese Adeyeye, Ehis Afeikhena, Akinola Akinmade, Babatunde Mustapha.

**Österreich:** *Paracelsus Medical University.* Jaroslav Presl, Patrick Rebnegger, Bjoern Rudisch, Gruenfelder Johanna, Rokitte Karin.

**Portugal:** *Centro Hospitalar Tondela.* Filipa M CorteReal, Jorge A Pereira, Joao L Pinheiro, Daniela M Pinto, Andreia J Santos, Andreia M Silva. *Hospital Garcia de Orta.* Susana Henriques, Joao Melo, António Miguel Pereira, Antonio Miguel Pereira. *Hospital da Horta, EPER.* Ana Margarida Cabral, Bruno Dias Couto, Barbara Nunes Gama, Catarina Santos Rodrigues. *Unidade Local de Saúde de Matosinhos - Hospital Pedro Hispano.* Mara Nunes, Bruno Ribeiro Silva, Daniela Tavares, Daniela Tavares.

**Romania:** *'Constantin Papilian' Emergency Clinical Military Hospital of Cluj-Napoca.* Toma Mihai, Oprea C Valentin.

**Serbia:** *General Hospital Vršac.* Srdjan S Putnik. *General Hospital Đorđe Joanović.* Petar Andjic, Marija Djujic, Rastislav Filko, Vanja Kunkin, Andjela Milak, Aleksandar Ognjenovic. *Zemun.* Nebojsa Mitrovic, Goran Aleksandric. *Medical Academy.* Mihailo Bezmarević, Sasa Dragović, Milan Jovanović, Bosko Milev, Miroslav Mitrović, Srdjan Petković. *Novi Sad.* Valentina Isakovic, Nikola Zoran Nikolic, Predrag Radic, Dragan Luka Vasic. *Clinical Center of Serbia.* Zlatibor M Loncar, Dusan D Micic, Vladimir R Resanovic, Pavle D Vladimir. *Clinical Center of Serbia; Medical Faculty, University of Belgrade.* Krstina S Doklestic Vasiljev. *University Clinical Hospital Center "Zvezdara" Clinic for Surgery "Nikola Spasic".* Ljiljana Velibor Milic, Vladica Velibor Cuk, Jovan Todor Juloski, Radisav Slavoljub Radulovic, Dragana Dragan Arbutina.

**Spain:** *Complejo Asistencial Universitario de Salamanca.* Jacobo Trebol, Manuel Torres-Jurado, Andres J Valera-Montiel, Francisco E Blanco-Antona. *Hospital Clínico Universitario.* Beatriz de Andrés-Asenjo, Maria Ruiz-Soriano, Tania Gómez-Sanz, Andrea Vázquez-Fernández, Juan Beltran de Heredia. *Hospital General Universitario Gregorio Marañón.* Cristina Rey-Valcárcel, Monica Ballón-Bordon, Maria Pérez-Díaz, Maria Dolores Sanchez-Rodriguez, Jose David Gonzalez-Esteban. *Hospital Nuestra Señora de Sonsoles.* Celia Alegre Nevado, Ricardo Montenegro Romero. *Hospital San Juan de Dios del Aljarafe.* Inés Capitán del Río, *Hospital Universitari Parc Taulí.* Andrea Campos-Serra, Raquel Gracia-Roman, Heura Llaquet-Bayo, Anna Muñoz-Campaña, Giulia Vitiello. *Hospital Universitario Donostia.* Lorena Apodaca Murguiondo, Inigo Augusto Ponce, Amaia Garcia Dominguez, Aintzane Lizarazu Perez. *Hospital Universitario Infanta Cristina.* Elena Sagarra Cebolla, Mónica García Aparicio, Paloma Garaulet González, Benito Miguel Josa Martínez, Miriam Fraile Vasallo. *Hospital Universitario J.* M Morales Meseguer. Mónica MengualBallester, Isabel Andrés Lucas Zamorano, Jose Martinez Moreno, Manuel Luis Buitrago Ruiz, Clara Piñera Morcillo. *Universitario Nuestra Señora de Candelaria.* Alberto Díaz García, Hanna Hernández Oaknin, Maria Pellicer Barreda, Jennifer Amparo García Niebla, Antonio Pérez Álvarez. *Hospital Universitario Príncipe de Asturias.* Diego Cordova, Laura Jiménez, Fernando Mendoza, Cristina Vera, Alberto Vilar Tabanera. *Hospital Universitario Virgen Macarena.* María de los Ángeles Gil-Olarte Márquez, José Antonio López-Ruiz, Mª Estela Romero-Vargas, Julio Reguera-Rosal, Alberto García-García, Beatriz Marenco de la Cuadra. *Hospital Universitario Virgen del Rocio.* Eduardo Perea del Pozo, Virginia Duran Muñoz, Felipe Pareja Ciuró. *Hospital de Mataró.* Ainoa Benavides dos Santos, Ernest Bombuy, Anna G-Monferrer, Sandra López Gordo. *Hospital de la Merced.* José Guerra, Vanessa Sojo, Begona De Soto, Aaron Roman. *Hospital del Mar.* Ana María González-Castillo, Elena Manzo, Estela Membrilla-Fernandez, Amalia Pelegrina-Manzano, Simone Cremona. *La Paz University Hospital.* Alexander Forero-Torres, Santiago Valderrabano, Francisco Reinoso Olmedo, Fuad Lopez Fernandez. *Urduliz Hospital.* Aitor Landaluce-Olavarria, Jon Barrutia- Leonardo, Alba Garcia-Trancho, Melania Gonzalez-De Miguel, Izaskun Markinez-Gordobil.

**United Arab Emirates:** *Sheikh Shakhbout Medical City.* Maryam Makki, Dana Altamimi, Sadhika Vinod.

**United Kingdom:** *Liverpool University Hospitals NHS Foundation TrustNHS.* Olga Rutka, John V Taylor. *Addenbrooke's Cambridge University Hospital.* M Denton, S Gourgiotis, R Ravi, A J Ribbits. *University Hospital Wishaw.* Jared Wohlgemut, Shehryar Rangana Khan, Christopher Leiberman, Sabreen P Elbakri, Charlie A Edgar. *Wirral University Teaching Hospitals NHS Foundation Trust.* Conor Magee, Oluwaseun Oyekan, Mehwish Ansar, Jeremy Wilson, Rahel Rashid.

**United States:** *Grand View Health.* Deborah Atwell, Joshua Cassedy, Brianna Gabriel, William Hoff, Shyam Murali. *University of Pennsylvania.* Anna E Garcia Whitlock, Carolyn Susman, Sarah Barnett, Emily Ertmann, Camden DeSanctis, Pavel Karasek, Nathan Klingensmith. *University of Texas Southwestern Medical Center.* Dale F Butler, Brandon Bruns, Ankeeta Mehta, Vanessa Nomellini, Keyus Patel, Anthony Tannous.

**Supplementary Results**

**Table S1 Comparison of population with any PROMS vs no PROM data**

| **Characteristic** | **PROMS Captured**  N = 756^1^ | **PROMS Missing**  N = 978^1^ | **p-value**^2^ |
| --- | --- | --- | --- |
| **Age** (years) | 69.0 (56.0, 79.0) | 69.0 (53.0, 80.0) | 0.4 |
| **Sex** |  |  | 0.2 |
| Female | 397.0 (52.5%) | 502.0 (51.3%) |  |
| Male | 359.0 (47.5%) | 472.0 (48.3%) |  |
| NA | 0.0 (0.0%) | 4.0 (0.4%) |  |
| **Comorbidities** |  |  | <0.001 |
| NA | 0.0 (0.0%) | 34.0 (3.5%) |  |
| No medical comorbidities | 182.0 (24.1%) | 184.0 (18.8%) |  |
| Yes | 574.0 (75.9%) | 760.0 (77.7%) |  |
| **Aetiology** |  |  |  |
| Adhesions | 424.0 (56.1%) | 556.0 (56.9%) |  |
| Hernia (primary inguinal/abdominal wall) | 119.0 (15.7%) | 110.0 (11.2%) |  |
| Tumor (carcinomatosis/metastatic deposit) | 27.0 (3.6%) | 89.0 (9.1%) |  |
| Hernia (incisional) | 47.0 (6.2%) | 56.0 (5.7%) |  |
| Hernia (internal) | 45.0 (6.0%) | 37.0 (3.8%) |  |
| Stricture (inflammatory) | 31.0 (4.1%) | 51.0 (5.2%) |  |
| Tumor (primary small bowel) | 13.0 (1.7%) | 30.0 (3.1%) |  |
| Hernia (parastomal) | 22.0 (2.9%) | 14.0 (1.4%) |  |
| Stricture (anastomotic) | 7.0 (0.9%) | 19.0 (1.9%) |  |
| Bezoar/Foreign body | 10.0 (1.3%) | 1.0 (0.1%) |  |
| Congenital (malrotation/Ladds band) | 5.0 (0.7%) | 5.0 (0.5%) |  |
| Gallstone ileus | 4.0 (0.5%) | 5.0 (0.5%) |  |
| NA | 0.0 (0.0%) | 4.0 (0.4%) |  |
| Endometriosis | 2.0 (0.3%) | 1.0 (0.1%) |  |
| **Bowel Ischemia** | 120.0 (28.0%) | 113.0 (30.2%) | 0.5 |
| **Prior non-operative management of SBO** | 154.0 (65.0%) | 215.0 (66.2%) | 0.8 |
| **Surgical intervention** | 466.0 (62.0%) | 418.0 (49.6%) | <0.001 |
| **Surgical complication** | 90.0 (12.1%) | 96.0 (12.2%) | >0.9 |
| ^1^Median (Q1, Q3); n (%) | | | |
| ^2^Wilcoxon rank sum test; Fisher's exact test; Pearson's Chi-squared test | | | |

**Table S2 Comparison of population with any PROMS vs no PROM data**

|  | **Appetite** | | | **Nausea** | | | **Bowels** | | | **Wellbeing** | | | **Overall** | | |
| --- | --- | --- | --- | --- | --- | --- | --- | --- | --- | --- | --- | --- | --- | --- | --- |
| **Characteristic** | **Beta** | **95% CI** | **p-value** | **Beta** | **95% CI** | **p-value** | **Beta** | **95% CI** | **p-value** | **Beta** | **95% CI** | **p-value** | **Beta** | **95% CI** | **p-value** |
| **Male Sex** | 4.8 | -0.80, 10 | 0.093 | 4.2 | -0.10, 8.5 | 0.055 | -3.8 | -9.4, 1.9 | 0.191 | 4.5 | -2.5, 12 | 0.208 | 6.7 | 1.0, 12 | 0.020 |
| **Comorbidities** | 0.72 | -6.7, 8.1 | 0.849 | -1.8 | -7.5, 3.9 | 0.531 | 0.67 | -6.8, 8.2 | 0.860 | -3.2 | -13, 6.2 | 0.507 | -6.6 | -14, 0.94 | 0.086 |
| **Prior non-operative SBO episode** | -7.3 | -13, -1.6 | 0.013 | 0.47 | -3.9, 4.9 | 0.836 | -2.6 | -8.4, 3.2 | 0.384 | -1.9 | -9.1, 5.3 | 0.605 | -1.5 | -7.3, 4.3 | 0.612 |
| **Surgical intervention** | 2.0 | -4.3, 8.2 | 0.539 | 5.9 | 1.1, 11 | 0.016 | 0.54 | -5.8, 6.8 | 0.868 | 6.0 | -1.9, 14 | 0.136 | 7.7 | 1.3, 14 | 0.018 |
| **Surgical Complication** | -7.3 | -17, 2.7 | 0.153 | -10 | -18, -2.3 | 0.010 | -5.2 | -15, 4.8 | 0.309 | -19 | -31, -6.2 | 0.003 | -13 | -23, -2.8 | 0.012 |
| **Aetiology** |  |  |  |  |  |  |  |  |  |  |  |  |  |  |  |
| Adhesions | — | — |  | — | — |  | — | — |  | — | — |  | — | — |  |
| Hernia | 3.2 | -4.0, 10 | 0.389 | 4.2 | -1.3, 9.7 | 0.137 | -0.22 | -7.5, 7.1 | 0.953 | 0.68 | -8.4, 9.7 | 0.884 | 3.3 | -4.1, 11 | 0.382 |
| Other | -3.9 | -15, 7.1 | 0.487 | -2.8 | -11, 5.6 | 0.513 | -0.66 | -12, 10 | 0.907 | -4.5 | -18, 9.4 | 0.523 | -1.7 | -13, 9.5 | 0.770 |
| Tumor | -9.0 | -25, 6.9 | 0.267 | -7.6 | -20, 4.7 | 0.225 | -3.9 | -20, 12 | 0.641 | -21 | -41, -0.44 | 0.045 | -8.4 | -25, 7.8 | 0.309 |

CI = Confidence interval

**Table S3: Summary of Summary of characteristics and scores according to water soluble contrast receipt.**

| Characteristic | No  N = 197^1^ | Yes  N = 157^1^ | p-value^2^ |
| --- | --- | --- | --- |
| **Treatment group** |  |  | <0.001 |
| *Non-operative management* | 56.0 (28.4%) | 114.0 (72.6%) |  |
| *Operated and complication* | 25.0 (12.7%) | 12.0 (7.6%) |  |
| *Operated no complication* | 116.0 (58.9%) | 31.0 (19.7%) |  |
| **Appetite** | 85.0 (65.0, 95.0) | 80.0 (65.0, 100.0) | 0.53 |
| **Bowels** | 91.7 (75.0, 100.0) | 91.7 (75.0, 100.0) | 0.41 |
| **Nausea** | 100.0 (87.5, 100.0) | 100.0 (87.5, 100.0) | 0.71 |
| **Wellbeing** | 75.0 (58.3, 100.0) | 75.0 (58.3, 100.0) | 0.86 |
| **Overall Bowel Function** | 80.0 (70.0, 90.0) | 80.0 (70.0, 90.0) | 0.70 |
| ^1^n (%); Median (Q1, Q3) | | | |
| ^2^Pearson's Chi-squared test; Wilcoxon rank sum test | | | |

**Table S4: Characteristics of matched groups comparing operative vs non-operative management of adhesional SBO**

| **Characteristic** | **N** | **No operation**  N = 143^1^ | **Operation**  N = 143^1^ | **p-value**^2^ |
| --- | --- | --- | --- | --- |
| **Age (years)** | 286 | 68.1 ± 15.7 | 64.9 ± 17.2 | 0.2 |
| **Sex** | 286 |  |  | 0.006 |
| Female |  | 64 (45%) | 87 (61%) |  |
| Male |  | 79 (55%) | 56 (39%) |  |
| **Comorbidities** | 286 |  |  | 0.018 |
| No medical comorbidities |  | 25 (17%) | 42 (29%) |  |
| Medical comorbidities |  | 118 (83%) | 101 (71%) |  |
| **Follow-up time (days)** | 286 | 128.5 ± 58.0 | 86.2 ± 57.5 | <0.001 |
| ^1^Mean ± SD; n (%) | | | | |
| ^2^Wilcoxon rank sum test; Pearson's Chi-squared test | | | | |

**Table S5: Characteristics of matched groups comparing open vs laparoscopic management of adhesional SBO**

| **Characteristic** | **N** | **Open operation**  N = 47^1^ | **Laparoscopic operation**  N = 47^1^ | **p-value**^2^ |
| --- | --- | --- | --- | --- |
| **Age (years)** | 94 | 64.6 ± 17.6 | 63.0 ± 17.8 | 0.6 |
| **Sex** | 94 |  |  | 0.7 |
| Female |  | 30 (64%) | 28 (60%) |  |
| Male |  | 17 (36%) | 19 (40%) |  |
| **Comorbidities** | 94 |  |  | >0.9 |
| No medical comorbidities |  | 18 (38%) | 18 (38%) |  |
| Medical comorbidities |  | 29 (62%) | 29 (62%) |  |
| **Follow-up time (days)** | 94 | 79.2 ± 55.2 | 104.7 ± 60.5 | 0.030 |
| ^1^Mean ± SD; n (%) | | | | |
| ^2^Wilcoxon rank sum test; Pearson's Chi-squared test; Wilcoxon rank sum exact test | | | | |

**Figure S1: Love plot for Operation vs no operation matching**


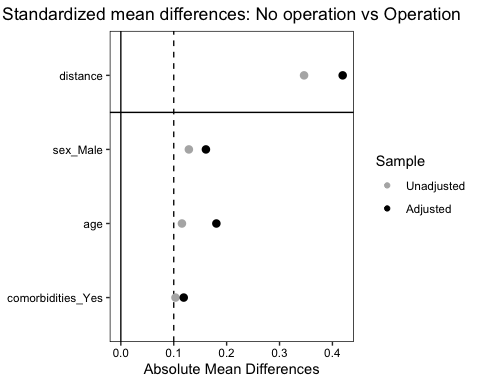


**Figure S2: Love plot for Laparoscopic vs open operation**


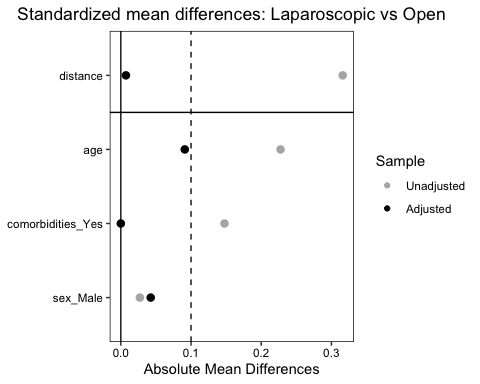

Supplement: Supplementary file 1 — Table S1. Comparison of population with any PROMS vs. no PROM data. Table S2. Comparison of population with any PROMS vs. no PROM data. Table S3: Summary of characteristics and scores according to water soluble contrast receipt. Table S4: Characteristics of matched groups comparing operative vs. non‐operative management of adhesional SBO. Table S5: Characteristics of matched groups comparing open vs. laparoscopic management of adhesional SBO. Figure S1: Love plot for operation vs. no operation matching. Figure S2: Love plot for laparoscopic vs. open operation. [file CODI-28-0-s001.docx]
